# Removable thermoplastic appliances modified by incisal cuts show altered biomechanical properties during tipping of a maxillary central incisor

**DOI:** 10.1186/s40510-017-0183-z

**Published:** 2017-08-28

**Authors:** Phillipp Brockmeyer, Katharina Kramer, Florian Böhrnsen, Rudolf Matthias Gruber, Sarah Batschkus, Tina Rödig, Wolfram Hahn

**Affiliations:** 1 Department of Oral and Maxillofacial Surgery, University Medical Centre Goettingen, Robert-Koch-Str. 40, 37075 Goettingen, Germany; 2 Department of Medical Statistics, University Medical Centre Goettingen, Humboldtallee 32, 37073 Goettingen, Germany; 3 Department of Preventive Dentistry, Periodontology and Cariology, University Medical Centre Goettingen, Robert-Koch-Str. 40, 37075 Goettingen, Germany; 4 Department of Orthodontics, University Medical Centre Goettingen, Robert-Koch-Str. 40, 37075 Goettingen, Germany; 5 Private practice, Goettingen, Germany

**Keywords:** Removable thermoplastic appliances, Biomechanics, Incisal cut

## Abstract

**Background:**

The present study aimed to evaluate the force delivery of removable thermoplastic appliances (RTAs), modified by different sized incisal cuts, during tipping of a maxillary central incisor in palatal and vestibular direction.

**Methods:**

Forty-five RTAs from three different materials (Biolon®, Erkodur®, Ideal Clear®) of the same thickness (1 mm) were used. Analysis was performed on a separated maxillary central incisor which was part of a resin model with a complete dentition. In 15 RTAs, of different material, a cut was inserted at the incisal edge of tooth 11. In 15 other appliances, the cut was extended to teeth 12 and 21. Fifteen aligners remained uncut. The experimental tooth was tipped starting from the zero position in 0.05° steps to a maximal deflection of ± 0.42° of the incisal edge in vestibular and palatal direction, after positioning the RTA onto the model.

**Results:**

The horizontal (Fx) and the vertical (Fz) force components were decreased by approximately half with increasing cut size. Fz values changed during palatal tipping from a weak intrusive force, for aligners without cut, to an extrusive force with increasing cut size. Compared to both other materials used (Erkodur® and Ideal Clear®), the Biolon® aligners showed significantly higher Fx and Fz values (*p* < 0.0001, respectively).

**Conclusions:**

RTAs modified by different sized incisal cuts show altered biomechanical properties and an inversion of the vertical force component, during tipping of a maxillary central incisor.

## Background

Removable thermoplastic appliances (RTAs) can be used in patients as a less visible alternative to conventional, fixed, orthodontic treatment appliances [1–4]. Minimal malocclusions, slight arch expansions, and corrections of a deep overbite are indication ranges [5]. Bodily tooth movements are restricted, as aligners primarily transfer forces produced by point contact between the appliance and the tooth [6, 7]. This results mainly in tipping and intrusive movements [6]. Forces required for tooth movement can be generated by local and whole body deformation of the aligner when it is placed onto the dental arch. This deformation results from a discrepancy between the actual and the intended tooth position incorporated into the appliance [7–9]. The appliance manufacturing process increases material stiffness by incorporating bends and edges into the aligner. This increase in stiffness is mainly located at the incisal edge [9]. Therefore, it might be appropriate to attenuate the generated force component by inserting cuts into the material in this region.

## Methods

**Fig. 1:**
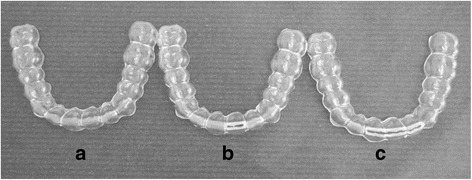
**a** Uncut aligner. **b** Aligner with incisal cut on tooth 11 (z11). **c** Aligner with incisal cut from tooth 12 to 21 (z12–21)

Force components were analysed using a measurement device containing a nano-17 sensor (ATI Industrial Automation, Apex, USA), as described in prior publications [7–11]. The sensor was connected to a separated maxillary central incisor as part of a standardized resin model (Frasaco GmbH, Tettnang, Germany). Analysis was carried out in an incubator at 37 °C. An impression (Tetrachrom®, Kanidenta, Herford, Germany) of the model with the measuring tooth placed on the measuring device was made. Subsequently, a plaster model (GC Fujirock® EP, GC GERMANY GmbH, Munich, Germany) was fabricated. Two model duplicates were produced by using Adisil® blau 9:1 (SILADENT Dr. Böhme & Schöps GmbH, Goslar, Germany). Following, an impression was taken from both models and five model duplicates were manufactured, as described above, respectively. Forty-five RTAs (15 for each material) with a similar extension of 2.5 mm beyond the gingival margin were produced from three different materials (Biolon®, Dreve Dentamid GmbH, Unna, Germany; Erkodur®, Erkodent® Erich Kopp GmbH, Pfalzgrafenweiler, Germany; Ideal Clear®, Dentsply GAC, Gräfelfing, Germany). For the thermoforming process of the Biolon® aligners, the Drufomat-TE appliance (Dreve Dentamid GmbH, Unna, Germany) was used. Ideal Clear® appliances were prepared with the ‘Vacuum Forming Machine’ 202 (Dentsply GAC, Gräfelfing, Germany) and Erkodur® aligners with the Erkoform RVE device (Erkodent® Erich Kopp GmbH, Pfalzgrafenweiler, Germany). Appliance edges were trimmed along the marginal sulcus with HSS twist drills and Lisko-S polishing discs (Erkodent® Erich Kopp GmbH, Pfalzgrafenweiler, Germany). The resulting forces were evaluated in 15 aligners without cut, 15 aligners with an incisal cut on the measuring tooth 11 (z11; the incisal edge of tooth 11 was exposed), and 15 aligners with an incisal cut from tooth 12 to 21 (z12–21) (Fig. 1). To produce cuts of the same size, their length and depth were marked using a ruler and a pen. These cuts were again prepared by HSS twist drills and Lisko-S polishing discs. The inner surface of the aligners was moistened using artificial saliva (University Pharmacy, Goettingen, Germany). Before analysis, all forces were set to zero. With the aligner in place, the experimental tooth was tipped in vestibular and palatal direction from 0° to 0.42° (24.9 arcminutes) and back to 0° in 0.05° (2.7 arcminutes) steps. Data was recorded five times after each movement. To protect the sensor from overloading, the incisal edge was maximally deflected up to ± 0.151 mm from the initial position in all cases. This activation range is comparable to the lowest activation range value documented in the literature for thermoplastic aligner systems [12].

### Statistical analysis

Data was analysed using a mixed, multifactorial analysis of variance (ANOVA) with repeated measurements. Since there was no normal distribution, observation ranks were used. All tests were performed at a significance level of *α* = 5%, using the statistical software SAS (SAS Institute Inc., Cary, NC/USA).

## Results

### Horizontal force component

#### Uncut aligners

Regardless of the tipping direction, the highest horizontal force component (Fx) values were observed for the Biolon® aligners; the lowest values were found during palatal tipping for the Erkodur® and vestibular tipping for the Ideal Clear® aligners (Table 1). Mean values during palatal tipping ranged from 2.56 N (SD 0.57 N) to 4.03 N (SD 0.36 N) and during vestibular tipping from 3.05 N (SD 0.80 N) to 5.41 N (SD 0.74 N).

**Table 1:** Means and standard deviations (SD) of the horizontal force component (Fx) for modified aligners (z11 = cut on tooth 11; z12–21 = cut from tooth 12 to 21) and uncut (none) aligners during palatal and vestibular tipping (± 0.151 mm deflection distance) for all three materials used

Tipping range	Material thickness	Material	Cut	N	Mean (N)	SD (N)
−0.151 mm palatal	1 mm	Biolon®	None	5	4.03	0.36
		Erkodur®	None	5	2.56	0.57
		IdealClear®	None	5	2.91	0.42
		Biolon®	z11	5	3.0	0.40
		Erkodur®	z11	5	1.94	0.48
		IdealClear®	z11	5	2.28	0.20
		Biolon®	z12–21	5	1.70	0.12
		Erkodur®	z12–21	5	1.49	0.25
		IdealClear®	z12–21	5	1.48	0.59
+0.151 mm vestibular	1 mm	Biolon®	None	5	5.41	0.74
		Erkodur®	None	5	3.11	0.23
		IdealClear®	None	5	3.05	0.80
		Biolon®	z11	5	4.07	0.26
		Erkodur®	z11	5	2.47	0.74
		IdealClear®	z11	5	2.78	0.55
		Biolon®	z12–21	5	2.09	0.40
		Erkodur®	z12–21	5	1.75	0.33
		IdealClear®	z12–21	5	1.72	0.89

#### Aligners with cut on the incisal edge of tooth 11 (z11)

Table 1 summarises the Fx values for z11 at a deflection of ± 0.151 mm. The lowest values were measured during both tipping directions for the Erkodur® aligners. The highest values were found for the Biolon® appliances. Mean values during vestibular tipping ranged from 2.47 N (SD 0.74 N) to 4.07 N (SD 0.26 N) and during palatal tipping from 1.94 N (SD 0.48 N) to 3.0 N (SD 0.40 N).

#### Aligners with cut on the incisal edge from tooth 12 to 21 (z12–21)

Table 1 shows the mean Fx values and SD for z12–21 at a deflection of ± 0.151 mm. The highest values could be observed for the Biolon® aligners, whereas the lowest values were found for the Ideal Clear® appliances. Overall, the force values during vestibular tipping were higher than those during palatal tipping. Mean values during palatal tipping ranged from 1.48 N (SD 0.59 N) to 1.70 N (SD 0.12 N) and during vestibular tipping from 1.72 N (SD 0.89 N) to 2.09 N (SD 0.40 N).

### Correlations and interactions between material, deflection distance, and incisal cut for Fx

**Fig. 2:**
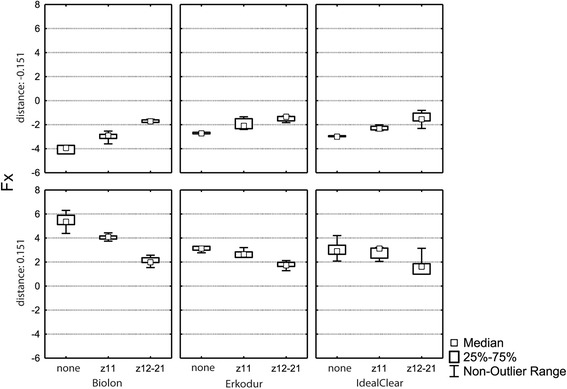
Horizontal force component (Fx) for the respective deflection distance (± 0.151 mm) separately for all three materials used (Biolon®, Erkodur®, Ideal Clear®) depending on the respective cut (none, z11, z12–21)

The ANOVA revealed a significant effect of the deflection distance (*p* < 0.0001), the material (*p* < 0.0001) and the incisal cut (*p* < 0.0001) on the Fx component. A significant interaction between the material and the incisal cut was found (*p* = 0.0429). No statistically significant interactions between the deflection distance and the material (*p* = 0.6479), the deflection distance and the incisal cut (*p* = 0.4201), and the deflection distance and the material and the cut (*p* = 0.8381) could be observed. Since the analysis revealed a significant interaction between the material and the cut, analysis was performed separately for each material (Biolon®, Erkodur®, Ideal Clear®, respectively) (Fig. 2).

#### Biolon®

A statistically significant correlation between the deflection distance (*p* < 0.0001) and the incisal cut (*p* < 0.0001) could be observed. No significant interaction between the deflection distance and the cut was found (*p* = 0.1995).

#### Erkodur®

A significant effect of the deflection distance (*p* < 0.0107) and the incisal cut (*p* < 0.0001) was found. No interaction between the deflection distance and the incisal cut (*p* = 0.7348) could be observed.

#### Ideal Clear®

No significant effect of the deflection distance (*p* < 0.2087) could be observed. The analysis revealed a significant influence of the incisal cut (*p* < 0.0001) on the measured Fx component. The interaction between deflection distance and the incisal cut was not significant (*p* = 0.6635).

### Vertical force component

#### Uncut aligners

Mean Fz values during palatal and vestibular tipping are summarized in Table 2. Overall, Fz values were higher during vestibular than palatal tipping. Regardless of the tipping direction, the highest Fz values were measured for the Biolon® aligners. The lowest values were found during palatal and vestibular tipping for the Ideal Clear® aligners. Mean values during palatal tipping ranged from − 0.02 N (SD 0.22 N) to − 0.16 N (SD 0.13 N) and during vestibular tipping from − 1.0 N (SD 0.29 N) to − 2.55 N (SD 0.46 N). The sign of Fz values reflects the effective force direction. A negative sign indicates an intrusive Fz, whereas positive values indicate an extrusive Fz component.

**Table 2:** Mean values and standard deviations (SD) of the vertical force component (Fz) for modified aligners (z11 = cut on tooth 11; z12–21 = cut from tooth 12 till 21) and uncut (none) aligners during palatal and vestibular tipping (± 0.151 mm deflection distance) for all three materials used

Tipping range	Material thickness	Material	Cut	N	Mean (N)	SD (N)
−0.151 mm palatal	1 mm	Biolon®	None	5	−0.16	0.13
		Erkodur®	None	5	0.03	0.19
		IdealClear®	None	5	−0.02	0.22
		Biolon®	z11	5	0.19	0.59
		Erkodur®	z11	5	0.11	0.17
		IdealClear®	z11	5	0.10	0.35
		Biolon®	z12–21	5	0.51	0.16
		Erkodur®	z12–21	5	0.32	0.25
		IdealClear®	z12–21	5	0.26	0.33
+0.151 mm vestibular	1 mm	Biolon®	None	5	−2.55	0.46
		Erkodur®	None	5	−1.13	0.25
		IdealClear®	None	5	−1.00	0.29
		Biolon®	z11	5	−0.69	0.31
		Erkodur®	z11	5	−0.65	0.17
		IdealClear®	z11	5	−0.58	0.20
		Biolon®	z12–21	5	−0.35	0.18
		Erkodur®	z12–21	5	−0.48	0.19
		IdealClear®	z12–21	5	−0.37	0.33

The sign of Fz values reflects the effective force direction. A negative sign indicates an intrusive Fz, whereas positive values indicate an extrusive Fz

#### Aligners with cut on the incisal edge of tooth 11 (z11)

The lowest Fz values at a deflection distance of − 0.151 mm were observed for the Erkodur® aligners; whereas for a deflection distance of + 0.151 mm, the lowest values were measured for the Ideal Clear® appliances. The highest Fz values were found independent of deflection direction for the Biolon® aligners. Mean values during vestibular tipping ranged from − 0.58 N (SD 0.20 N) to − 0.69 N (SD 0.31 N) and during palatal tipping from 0.10 N (SD 0.35 N) to 0.19 N (SD 0.59 N).

#### Aligners with cut on the incisal edge of teeth 12 to 21 (z12–21)

The highest Fz values at a deflection distance of − 0.151 mm were measured for the Biolon® aligners; the lowest values were found for the Ideal Clear® appliances. For a deflection distance of + 0.151 mm, the analysis revealed the lowest values for the Biolon® and the highest for the Erkodur® aligners. Mean values during vestibular tipping ranged from − 0.35 N (SD 0.18 N) to − 0.48 N (SD 0.19 N) and during palatal tipping from 0.26 N (SD 0.33 N) to 0.51 N (SD 0.16 N).

### Correlation and interactions between materials, deflection distance, and incisal cut for Fz

**Fig. 3:**
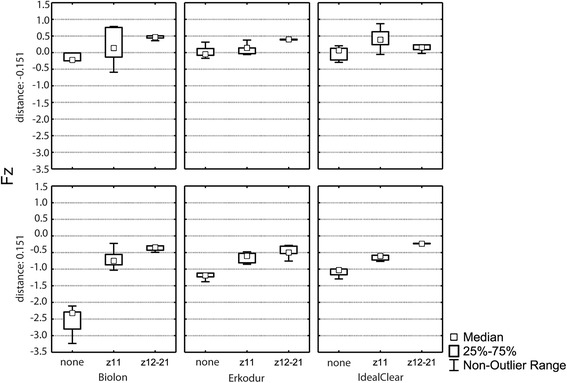
Vertical force component (Fz) at the respective deflection distance (± 0.151 mm) separately for all three materials used (Biolon®, Erkodur®, Ideal Clear®) depending on the respective cut (none, z11, z12–21)

The ANOVA revealed statistically significant effects of the deflection distance (*p* < 0.0001) and the incisal cut (*p* < 0.0001) on the Fz component. No significant effect of the material (*p* = 0.4672) was found. In addition, no significant interactions between the deflection distance and the material (*p* = 0.8743), the deflection distance and the incisal cut (*p* = 0.3741), the material and the incisal cut (*p* = 0.1590), and the deflection distance and the material and the cut (*p* = 0.6148) could be observed (Fig. 3).

## Discussion

The measuring device used in the present investigation is comparable to the one already described previously [13–17]. A rigid connection between measuring tooth and sensor was used. So far, it has been impossible to simulate the periodontal ligament (PDL) using a measuring device [18, 19]. Therefore, the measured forces must be interpreted as an immediate and transient sequence, as no significant tooth movement can be expected at this point due to the PDL’s visco-elastic properties [20, 21].

The results confirm a direct dependency between the aligner material and the magnitude of force delivery, as has been described in preliminary investigations [7, 9–11]. The Fx and Fz values for the Biolon® aligners were significantly higher than those for the two other materials tested (Erkodur® and Ideal Clear®). Apart from different material properties comprising individual elastic moduli [22], the manufacturing process plays a crucial role in force delivery strength. The Biolon® appliances were prepared by using 6 bar overpressures; while in the Erkodur® and Ideal Clear® appliances, a 0.8 bar under pressure was used. Thermoforming using high positive overpressure leads to more precise fitting of the appliance on the individual oral structures. Depending on the degree of better play of the appliance in the anterior region, where a tooth is tipped, complete deformation of the aligner results. Superior fit and, consequently, higher friction generate higher restoring force components. A space holder foil of 0.05 mm was additionally used for Erkodur® appliance preparation. Friction was decreased, resulting in a reduced force application compared to the two other test materials.

**Fig. 4:**
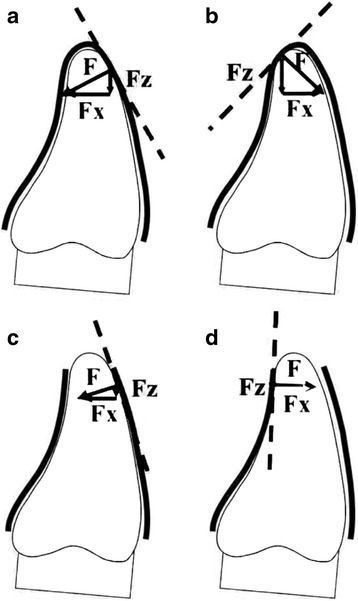
When a tooth is tipped, depending on the degree of misfit between tooth and appliance, a local elastic deformation of the appliance material at the point of contact with the respective restoring force results. The appliance is lifted off at the anterior part and is held in place at the posterior end depending on retention. Thus, a whole-body deformation of the appliance also creates a restoring force. Both restoring forces are acting vestibular and palatal on differently inclined inner surfaces of the appliance (**a**, **b**). This results in different Fx and Fz values. When the appliance is cut at the incisal edge, the inclined inner surfaces are partly removed and the local deformation of the appliance is located more apically at more vertically orientated appliance surfaces (**c**, **d**). This means, if the incisal link between the vestibular and the palatal appliance wall is detached, during tipping, the appliance walls can be moved more horizontally with less restoring force, depending on the extension of the cut. Thus, the appliance is lifted off much lesser in the anterior region which, in consequence, reduces the vertical force component again significantly (**c**, **d**). Depending on the inclination of the inner wall of the aligner at the contact area between appliance and tooth with incisor-side of cut aligners, even a reversal of the force component Fz (extrusion) may be observed

The incisally cutted aligners exhibited reduced values for the horizontal (Fx) and the vertical (Fz) force components with increasing cut size. As has been described before [9], an important component of the high force values occurring during tooth movements using aligners is caused by the increase in material stiffness due to incorporating bends and edges into the appliance during thermoforming. When the appliance is cut at the incisal area, this stiffening is reduced. This could be due to an altered stress–strain relationship in the modified aligners. With increasing cut size, the aligner could be warped more easily near the tooth contact area (below the incisal edge). This results in less force against the tooth to be treated (Fig. 4).

For the first time, the present investigation demonstrated a possible inversion of the Fz component during palatal tipping as Fz values changed from a weak intrusive force for aligners without cut to an extrusive force for those with a cut of increasing size. One reason for this might be the different morphology of the vestibular (convex) and palatal (both convex and concave) surfaces of the experimental tooth, resulting in a unique interaction between inclination and shape of the interior aligner surface and the anatomy of the tooth in the contact area during force generation. The contact point between appliance and tooth, where the force is generated in uncut aligners, is located near the incisal edge at an inclined inner surface of the appliance [10, 11]. During tipping, this inclined surface results in a single point force application. With incisal cut, the aligner material can move more horizontally at the contact area. In this case, the force components that are generated are less at an inclined surface, which results in reduced intrusive force values (Fz). What is more, during palatal tipping, the incisal parts of the tooth slip relatively in an apical direction on the palatal aligner surface with high friction. This could explain the extrusive Fz values (Fig. 4). Compared to uncut aligners, the material at the area around the contact point of appliances with a cut can be more deformed with lower resulting forces.

The amount of orthodontically induced inflammatory root resorptions has been shown to be directly correlated with the force magnitude applied [23–25]. By using removable aligners, less inflammatory root resorptions are caused, even with higher forces [26], which have also been reported by Barbagallo and colleagues during premolar tipping [27]. Therefore, modifying the aligner by an incisal cut could reduce even more the risk of orthodontically induced inflammatory root resorptions.

## Conclusions

The conclusions of the study are as follows:

- Removable thermoplastic aligners modified by incisal cuts exhibit altered biomechanical properties and a possible inversion of the vertical force component.
- Incisal cuts decrease the horizontal and vertical force components significantly.
- This could be used to reduce the number of aligners during clinical application.
